# Attentional Modulation and Selection – An Integrated Approach

**DOI:** 10.1371/journal.pone.0099681

**Published:** 2014-06-25

**Authors:** Albert L. Rothenstein, John K. Tsotsos

**Affiliations:** Department of Electrical Engineering and Computer Science, and Centre for Vision Research, York University, Toronto, Canada; Centre de Neuroscience Cognitive, France

## Abstract

Various models of the neural mechanisms of attentional modulation in the visual cortex have been proposed. In general, these models assume that an ‘attention’ parameter is provided separately. Its value as well as the selection of neuron(s) to which it applies are assumed, but its source and the selection mechanism are unspecified. Here we show how the Selective Tuning model of visual attention can account for the modulation of the firing rate at the single neuron level, and for the temporal pattern of attentional modulations in the visual cortex, in a self-contained formulation that simultaneously determines the stimulus elements to be attended while modulating the relevant neural processes.

## Introduction

While visual scenes typically contain multiple objects, the capacity of the visual system to process all these objects at the same time is limited [1]–[4]. In experimental settings, when presented with multiple objects, subjects' performance decreases and typical errors are present [5]–[8]. Stimuli are said to compete for neural representation, and the mechanisms of this competition and their modulatory effect on neural responses have been a subject of intense investigation and modeling.

There have been several models of visual attentive neural modulations, and all assume that an external ‘attention’ parameter is provided by some other neural process. Its value as well as the selection of neuron(s) to which it applies are assumed, but its source and how all of this is determined are unspecified. Our theory, Selective Tuning (ST), presents a novel formulation that solves these problems [9]. In ST, neuron response is the result of attentive modulation of its inputs across time from the whole network involving feedforward, recurrent, and lateral interactions. Although the Selective Tuning model has been presented previously in several venues, in this paper we, for the first time, take its basic equations, refine and deploy them for the task of simulating single neuron responses in attentive and non-attentive experimental situations, following classic single neuron recording results in the literature. We show that not only does ST provide very good comparisons to single neuron firing rates in attentive tasks but also show how, given visual stimuli, the stimulus locus of attention is computed and used throughout the network. The goal of this paper is to show how this model captures the essence of attentive modulation as well as its competitors, while additionally adding the critically missing element of attentional computation. Importantly, ST goes beyond other models in defining a modulatory mechanism at a finer level of abstraction than previously accomplished; other aspects of ST are described elsewhere [9].

The paper starts with a brief overview of ST and a review of computational models of attentional modulation. The microcircuitry of ST and the equations that govern its behaviour are described next, followed by two computational modeling experiments. We describe the experiments that form the foundation for the biased competition theory [10]–[11], followed by a simulation of these experiments using ST. Additionally, the temporal latency of selective attention modulation across areas in ST is presented and compared to that of the macaque visual system [12]. These experiments demonstrate the fact that ST can account for the modulation of the firing rate at the single neuron level, and for the temporal progression of attentional modulations in the visual cortex in a self-contained formulation without the external attentional parameter. The paper concludes with a comparison of the specifics of the models considered, analyzing relative strengths and weaknesses.

### Models of Attentive Neural Modulation

A wide variety of models have been proposed to explain the attentional modulation of neural activations, in this section we briefly review a representative subset. The descriptions included below and the equations provided in Appendix S1 are meant mainly to illustrate the wide variety of solutions proposed, and to highlight the way attentional modulation is implemented, rather than being exhaustive descriptions of the models. For full details, complete sets of equations and biological justification, the reader is referred to the original sources. For each of the models, there is no assessment of their actual results presented because each in its own way shows good matches to data and/or behavior. As a result, the point of this comparison is to clarify commonalities, differences, gaps, and strengths. Here we will focus on how the different models present attentional neural modulation. For general reviews of theories of attention see [13]–[15], [9], and specifically for computational modeling, see [16]–[18].

The biased competition theory [10], [11] proposes that neurons representing different features compete and that attention biases this competition in favor of neurons that encode the attended stimulus. The Biased Competition model [19] has been proposed as a demonstration of the biased competition theory. Attention is assumed to increase the strength of the signal coming from the inputs activated by the attended stimulus, implemented by increasing the associated synaptic weights. The model makes no claims of biological plausibility – the equations are not good fits for neural responses, no actual competition is implemented, and there is no known mechanism for the multiplicative modulation of synaptic weights [20].

A significant number of other models of biased competition have tried to build upon the Biased Competition model, by filling in the missing biologically-plausible mechanism, by including additional neuron types, and by extending the model to full networks.

The Neurodynamical model [21] is a large-scale implementation of biased competition that consists of several interconnected network modules simulating different areas of the dorsal and ventral path of the visual cortex. Each module consists of a population of cortical neurons arranged in excitatory and inhibitory pools. The inhibitory pool receives excitatory input from all the excitatory pools and provides uniform inhibitory feedback to each of the excitatory pools, thus mediating competition between them. The temporal evolution of the system is described within the framework of a mean-field approximation, i.e. an ensemble average of the neural population is calculated in order to obtain the corresponding activity. The model asserts that feature attention biases intermodular competition along the ventral pathway (simulated areas V4 and IT), and spatial attention biases intermodular competition along the dorsal pathway (simulated areas V1, V4 and PP).

The Feedback Model of Visual Attention [20] improves on the Biased Competition model by providing a biologically-justified mechanism and microcircuitry for input modulation. Compared to most other implementations of biased competition, in which neurons compete by inhibiting each other's output, in the Feedback Model of Visual Attention neurons compete by laterally inhibiting other neurons' inputs. The key observation that drives the model is that feedforward connections seem to be primarily made in basal dendrites, while feedback connections preferentially target apical dendrites, thus appearing to have functionally different roles.

The reentry hypothesis [22] models top-down modulation as a gain control mechanism on the input feedforward signal, multiplicatively increasing activations that match top-down predictions. The multiplicative gain allows for multiple forms of attentional selection, by combining signals originating from different areas (e.g. memory for stimulus-specific features, motor maps for location specific feedback) that allow the system to simulate a variety of experimental tasks.

An alternative account for attentional modulation comes in the form of the Feature Similarity Gain theory [23], [24], according to which attention can both enhance and reduce neural activations in proportion to the similarity between the attended stimulus and the preferred stimulus of the neuron. The attentional gain effect on neuronal responses is a graded function of the difference between the attended feature and the preferred feature of the neuron, independent of the stimulus. In the computational model of feature similarity gain proposed by [25], the neural response is described as a divisive contrast normalization process. A purely feature-based gain factor is independent of the spatial focus of attention and the properties of the visual stimulus.

Saliency based models [26] deal with the selection of stimuli, focusing on modeling eye fixation patterns, and generally do not address single neuron modulation. Attentional modulation is investigated in a model combining saliency and object recognition [27]. A modulation mask is obtained by rescaling the attentional selection field to the resolution of the layer where attention is to be applied (corresponding to visual areas V1 or V4), and neural activity is modulated by applying this mask with a modulation strength parameter, resulting in the suppression of neural activity outside of the attended spatial region. The impact of changing the modulation strength parameter on object recognition performance is investigated in detail.

Yet another class of models start from the observation that the neural representation of multiple concurrent stimuli is equivalent to a normalization [28], [29]. As normalization is viewed as a fundamental, canonical neural computation, the authors hypothesize that attention has an impact on neural activations by influencing the normalization process.

The Normalization Model of Attention [30] is an attempt to unify under a single computational model disparate results that are consistent with attention as a multiplicative gain factor, as a change in contrast gain, a sharpening of neural tuning, and various forms of attenuation and enhancement. The proposed model combines neural selectivity (termed “stimulus drive”) with an external “attention field” and a “suppressive field”, that pools activations corresponding to non-preferred stimulus and unattended locations, which is used as in normalization. An attentional gain applied before normalization accounts for the wide range of behaviours exhibited by the model.

The Normalization Model of Attentional Modulation [31] proposes that the primary effect of attention is to modulate the strength of normalization mechanisms by using different nonlinear summation regimens.

The attentional modulation of firing rate and synchrony in a biophysical network of spiking neurons is the subject of the Cortical Microcircuit for Attention model [32]. Attention was modeled as a change in the driving current to the network neurons. In addition to excitatory neurons, the investigation focuses on the role of interneurons, and suggests that both feedforward and top-down interneurons play a role. These are differentially modulated by attention: the firing rate of the feedforward interneurons increases with spatial attention and decreases with feature-based attention, whereas the top-down interneurons increase their firing rate with feature-based attention and shift the network synchrony from the beta to the gamma frequency range. Based on the model, the authors propose a canonical circuit for attention, and present a number of concrete and testable predictions.

An example of a modeling effort that tries to reconcile different approaches is the integrated microcircuit model of attentional processing [33]. The integrated microcircuit model is a biophysically based network model of spiking neurons composed of a reciprocally connected loop of two (sensory and working memory) networks. A wide variety of physiological phenomena induced by selective attention are shown to naturally arise in such a system. The proposed neural circuit is an instantiation of feature-similarity gain modulation [23], [24].

Attention is modeled as a top-down signal originating in a working memory area, and primed by a cue at the start of the simulation.

Predictive coding [34] is reformulated as a form of biased competition [35], [36]. Every processing stage in the proposed model consists of prediction and error detection nodes, with firing rate dynamics defined by the prediction error. A nonlinear version of these equations, that obtains better fits to experimental data, is also presented.

Although all the models have good results, the source of the ‘attention’ parameter takes various forms, e.g.: ventral and dorsal prefrontal areas provide the external top-down bias that specifies the task [21], top-down signals corresponding to stimulus expectations or feedback processes generated by recurrent connectivity [20], prefrontal memory circuits [22], [31], and unspecified in some models.

The value of the attentional signal as well as the selection of neuron(s) to which it applies are generally assumed, and its source and the selection mechanism are unspecified. As a result, none of these models can address issues related to temporal relationships between stimuli and attentional modulation, and hierarchical communication. Also, because each model exhibits good performance, there is little that would allow one to decide which is correct. By demonstrating ST performance on a larger experimental set, we hope to resolve this problem.

### The Selective Tuning Model of Visual Attention

#### Basics of Selective Tuning

The Selective Tuning (ST) model of visual attention [37]–[39], [9] starts from ‘first principles’ and features a theoretical foundation of provable properties based on the theory of computational complexity [4], [40]–[42]. The ‘first principles’ arise because vision is formulated as a search problem (given a specific input, what is the subset of neurons that best represent the content of the image?) and complexity theory is concerned with the cost of achieving solutions to such problems. This foundation suggests a specific biologically plausible architecture as well as its processing stages. Research on ST has been driven by the desire to create a theory with strong neurobiological predictive power as well as utility in practice. The model has been implemented and tested in several labs applying it to guide computer vision and robotics tasks. It has also made a number of true predictions in its early papers that now have substantial behavioral and neurophysiologic experimental support (detailed in [9]).

ST is characterized by the integration of feedforward and feedback pathways into a network that is able to take high level decisions, and, through a series of response-based decision processes, identify the neurons that have participated in that decision. The ST feedback process does not rely on a spatial spotlight, so ST is able to select all parts of a stimulus, even if they do not share a location (e.g. stimuli with discontinuities due to overlap, or stimuli that are separated spatially due to the nature of the cortical feature maps).

The visual processing architecture is pyramidal in structure, as in other models (e.g. [43], [44]) with units within this network receiving both feed-forward and feedback connections. A pyramidal representation is a layered representation characterized by successively coarser spatial representations. When a stimulus is presented to the input layer of the pyramid, it activates in a feed-forward manner all of the units within the pyramid with receptive fields (RFs) mapping to the stimulus location; the result is a diverging cone of activity within the processing pyramid. It is assumed that response strength of units in the network is a measure of goodness-of-match of the stimulus within the receptive field to the model that determines the selectivity of that unit.

Selection relies on a hierarchy of Branch-and-Bound decision processes. Branch-and-Bound is a classic mechanism that is used in optimization problems [45] and recursive pruning within the branch-and-bound strategy is especially useful for a hierarchical system, such as ours. Our decision processes are implemented as θ-WTA, a unique form of the common winner-take-all algorithm, a parallel algorithm for finding the maximum value in a set. There is no single winner; rather response values are partitioned into ordered groups where partition bins have width θ. All neurons that have responses within the first bin (ie., largest responses within θ of each other in value) are selected as the winners. Winner-take-all competitions are commonly used, and generally accepted as a neurobiologically plausible component of visual attention models [46], [47]. To accomodate the needs of visual attention, models have used different multi-winner variants of the basic WTA algorithm, such as softMAX [48], θ-WTA [39] and k-WTA [49], each standing as a distinct prediction of its respective model.

In the first step of the algorithm, a θ-WTA process operates across the entire visual field at the top layer where it computes the global winner, i.e., the set of units with largest response. The θ-WTA can accept guidance to favor areas or stimulus qualities if that guidance is available but operates independently otherwise. The search process then proceeds to the lower levels by activating a hierarchy of θ-WTA processes. The global winner activates a θ-WTA that operates only over its direct inputs to select the strongest responding region within its receptive field. Next, all of the connections in the visual pyramid that do not contribute to the winner are pruned (inhibited). The top layer is not inhibited by this mechanism. However, as a result, the input to the higher-level unit changes and thus its output changes. This refinement of unit responses is an important consequence because one of the important goals of attention is to reduce or eliminate signal interference [4]. By the end of this refinement process, the output of the attended units at the top layer will be the same as if the attended stimulus appeared on a blank field. This strategy of finding the winners within successively smaller receptive fields, layer by layer, in the pyramid and then pruning away irrelevant connections through inhibition is applied recursively through the pyramid. The end result is that from a globally strongest response, the cause of that largest response is localized in the sensory field at the earliest levels. The paths remaining may be considered the pass zone of the attended stimulus while the pruned paths form the inhibitory zone of an attentional beam. The θ-WTA does not violate biological connectivity or relative timing constraints. This algorithm is hinted at by [50]: “[I]f the relevance of a stimulus feature depends on its context, any influences that attention may have on cells that respond to that feature will arrive to those cells after analysis of the context that signals the relevance of the feature. The time taken by that analysis will be reflected by a relatively long latency of attention-modulated cell responses to the relevant feature.”

In more neural terms, ST uses recurrent tracing of connections to achieve localization. The idea of tracing back connections in a top-down fashion was present in Fukushima's NeoCognitron model [43] and suggested even earlier by Milner [51]. Within the Selective Tuning model, whose earliest descriptions are found in [52], [37], [38], with accompanying details and proofs in [39]. It also appeared later in the Reverse Hierarchy Model [53], [54]. Only NeoCognitron and Selective Tuning provide realizations; otherwise, the two differ in all details. Fukushima's model included a maximum detector at the top layer to select the highest responding cell, and all other cells were set to their rest state. Only afferent paths to this cell are facilitated by action from efferent signals from this cell. In contrast, neural inhibition is the only action of ST, with no facilitation. The NeoCognitron competitive mechanism is lateral inhibition at the highest and intermediate levels. This lateral inhibition enhances the strongest single neurons thus assuming all spatial scales are represented explicitly, whereas ST finds regions of neurons, removing this unrealistic assumption. For ST, units losing the competition at the top are left alone and not affected at all — the non-attended visual world does not disappear as in NeoCognitron. ST's inhibition is only within afferent sets to winning units. This prediction of a space-limited suppressive surround firmly distinguishes the two approaches.

#### The Selective Tuning Circuit

**Figure 1:**
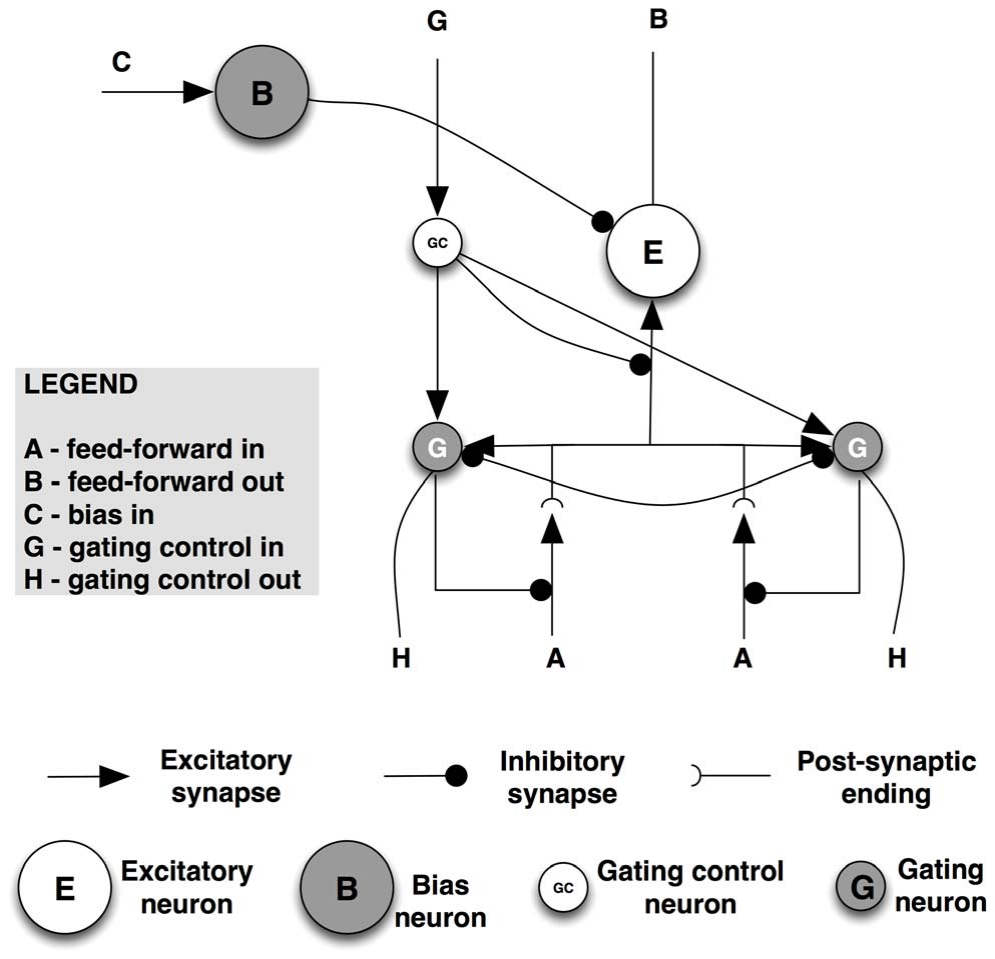
Selective Tuning microcircuit. Several types of neurons are required for ST to function. The connectivity among four classes of neurons — interpretive, bias, gating and gating control — is presented. The figure shows a single assembly that computes a single visual quantity (feature, object, etc.) at a single tuning profile.

Several types of neurons are required for ST to function. The connectivity among four classes of neurons — interpretive, bias, gating and gating control — is presented in Figure 1 (adapted from Figure 5.6 in [9]). The figure shows a single assembly that computes a single visual quantity (feature, object, etc.) at a single tuning profile. All elements of this single assembly represent computations at the same spatial location. At the same location, however, there are many such competing assemblies spanning the tuning ranges of all visual qualities.

Interpretive neurons are the classical feature-detecting neurons. They will be represented by *E*, and their activation by *e*. They receive feed-forward input from other areas that arrives in lamina 4 and provide an output to other areas from laminae 5 and 6.

Task information can be provided to the network by bias neurons. These provide top-down guidance for visual processing, whether the selection is for locations or regions in space, sub-ranges of visual features, objects, events, or whole scenes to attend to.

The gating sub-network, composed of gating and gating control neurons, is the major mechanism by which selection of attended neurons is accomplished and by which those neural activations are traced back down to their source, forming the path of the attentional beam. Their specific roles are described below.

We will use the following notation to represent connections between neurons:

⌅ represents the set of feed-forward connections to a neuron from all sources.

∨ represents the set of recurrent connections to a neuron from all sources.

Ξ represents the set of local connections to a neuron; that is, the neurons to which it is connected within the same visual area.

All these will be specialized by means of superscripts to distinguish the different kinds of connections. In order to keep the equations simple, we will assume that all activations and parameters correspond to a given assembly, and forgo indices that localize the neurons within the full network (but see [9] for the full formulation).

Given that our goal here is to understand how neural inputs and activations are modulated by attention, we will use a simple weighted sum of inputs formulation for the activation of neurons:
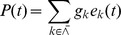
where g_k_ are weights specifying the strength of contribution from neuron k to the current neuron E and e_k_ is the activation of neuron k.

The neuron's firing rate S is defined by:
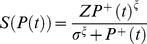
where Z is the maximum firing rate, *P^+^* the positive half-rectified value of *P*, the exponent ξ determines the maximum slope of the function (i.e., how sharp the transition is between threshold and saturation), and σ, the semi-saturation constant, determines the point at which S reaches half of its maximum. The value σ is determined by the base semi-saturation constant σ_0_ plus fast and slow after-hyperpolarizing potentials:
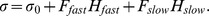



The fast (H_fast_) and slow (H_slow_) after-hyperpolarizing potentials are defined by:
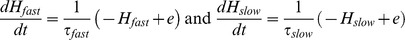
respectively. The effect of these variables is to slowly decrease the value of the neuron's activation *e* when the neuron is active.

The temporal variation of a neuron's response is governed by:
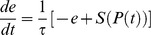
where *τ* is a decay time constant.

Bias inputs act by suppressing the input to task-irrelevant interpretive neurons. For any given neuron, the bias input is determined as the minimum value of all converging bias signals:
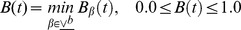
where ∨ ^b^ is the set of bias units making feedback connections to *E*. The default value of each bias unit is 1.0. Adding this bias to the neural response equation yields:
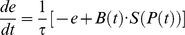



In ST, the input signals reaching a neuron are modulated by gating control signals that are a result of the winner-take-all competitions between activations. The gating sub-network is charged with determining the winning subset of the inputs to the pyramidal neuron *E*, suppressing feed-forward connections to *E* that correspond with the losers of the competition and transmitting the values of the gating neurons down to the next layer, to become the gating control signals for the next layer neurons.

The winner-take-all process creates an implicit partial ordering of the set of neural responses. The ordering arises because inhibition between units is not based on the value of a single neuron but rather on the difference between pairs of neural responses, where the difference must be at least as great as a task-specific parameter *θ, θ≥0.0*. This process is not restricted to converging to single values as it is in all other formulations; rather regions of neurons are found as winners. Competition depends on the difference between neuron response strengths: neuron *A* will inhibit *B* in the competition if *e_A_(t) – e_B_(t)>θ*. Otherwise, *e_A_* will not inhibit *e_B_*. Each input to the competition must be weighted by its role for the interpretive units that it feeds to reflect the importance of each input to the interpretive computation in the competition, Thus, the inputs to the gating network must be postsynaptic as shown in Figure 1. The θ-WTA process is defined by the recurrence relation:
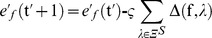
where *e′* and *t′* represent activation and time during the competition (i.e. competition starts at *t′ = 0*), and
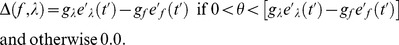



The gating control signals *ς* are defined as:
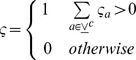
where *v^c^* is the set of gating control signals converging onto *E*. There is also one gating neuron, *γ_f_*, *f* ∈⌅, for each of the feed-forward inputs to *E*, 0≤*γ_f_* ≤1.0. This results in gating signals:
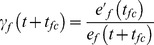



Integrating the gating signals into the ST equation, and dropping the time parameter ‘(t)’ for convenience, we obtain:
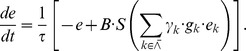



The complete ST equation also includes lateral cooperation signals, leading to the complete equation:
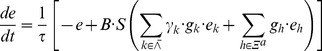
where −1.0≤*g_h_*≤1.0 is the weight of the connection from neuron *E_h_* to *E*, and *Ξ^a^* represent the connections horizontally across columns for neuron *E*. As these lateral signals are not relevant to the results presented here, the reader is referred to [9] for a full description. The performance of this model will be presented after the experimental setup is described.

## Results and Discussion

### Single-Neuron Attentional Modulation in the Macaque

**Figure 2:**
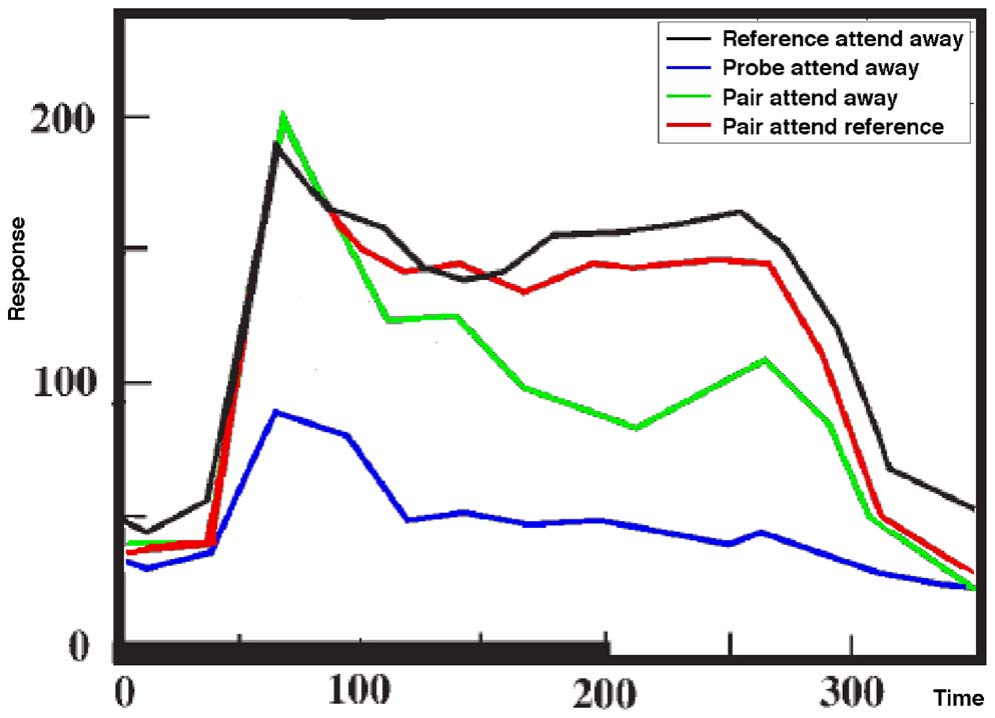
A summary of the Reynolds et al. experimental results. The experiment illustrates the basic attentional modulation effects, and consists of the presentation of one or two stimuli within a neuron's receptive field (RF), with attention directed to the area covered by the RF or away from it. When presented alone, one of the stimuli (the reference stimulus) elicits a strong response from the neuron – black line – while the other (the probe stimulus) elicits a weak response – blue line. When both stimuli are shown, and in the absence of attention, the presence of the probe results in a reduction of the neuron's response relative to the response to the reference stimulus alone – green line. With attention engaged and directed towards the reference stimulus, the response recovers, being similar to the response to the reference stimulus presented alone – red line.

The basic attentional modulation effects, a necessary starting point for any model, are presented by Reynolds et al. [19] and summarized in Figure 2. The experiment consists of the presentation of one or two stimuli within a neuron's receptive field (RF), with attention directed to the area covered by the RF or away from it. When presented alone, one of the stimuli (the reference stimulus) elicits a strong response from the neuron – black line in Figure 2, while the other (the probe stimulus) elicits a weak response – blue line. When both stimuli are shown, and in the absence of attention, the presence of the probe results in a reduction of the neuron's response relative to the response to the reference stimulus alone – green line. With attention engaged and directed towards the reference stimulus, the response recovers, being similar to the response to the reference stimulus presented alone – red line.

### Single-Neuron Attentional Modulation in ST

**Figure 3:**
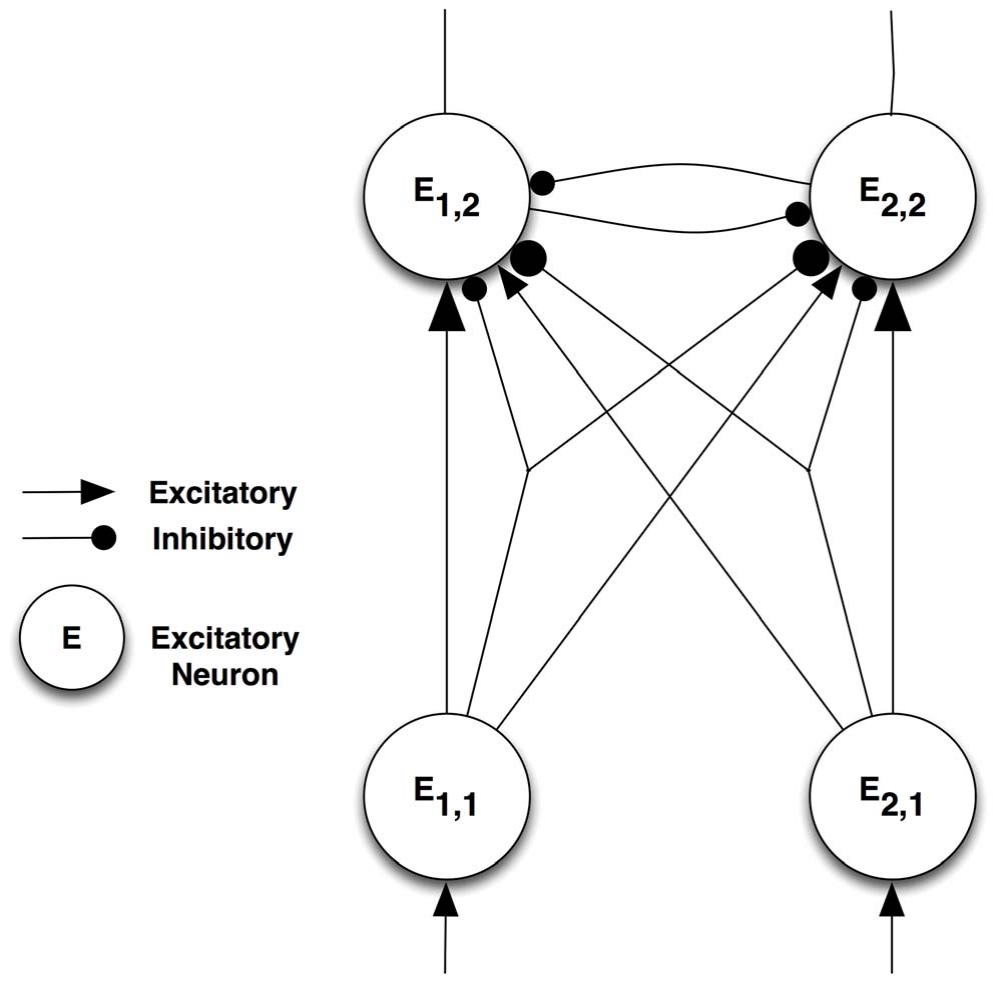
The network structure. Input neurons are at the bottom. Similar to the Reynolds and Desimone model [11], we include both excitatory and inhibitory inputs in all combinations. Excitatory and inhibitory connections are represented by arrows and circles, respectively. Connection size correlates with connection weight, i.e. E_1,2_ receives large inputs from E_1,1_ (excitatory) and E_2,1_ (inhibitory), and small inputs from E_1,1_ (inhibitory) and E_2,1_ (excitatory).

To investigate the modulation of neural activations due to ST attentional selection we used the simple circuit illustrated in Figure 3. The responses of the model neurons are defined by the ST equations presented above, the same equations are used for all 4 excitatory neurons, while the inhibitory interneurons are described by the same equations, omitting the gating components. Simulating the Reynolds and Desimone experiments [11], the two neurons at the bottom of the figure correspond to the reference (left, labeled E_1,1_) and probe (right, labeled E_2,1_) stimuli. The two neurons at the top of the figure represent neurons that have the reference (left, labeled E_1,2_) and the probe (right, labeled E_2,2_) as preferred stimulus. Their inputs and outputs correspond to the connections labeled A and B, respectively, in Figure 1. The excitatory input to the output units, represented by arrows is the sum of the activations of the input units multiplied by their respective weights (larger for the preferred stimulus, smaller for the non-preferred). Similarly, the inhibitory input is the weighted sum of the activations of two inhibitory units, one corresponding to each input. The θ-WTA competition between the two output units is represented as mutual inhibition. The model network also includes ST bias, gating and gating control units (with the associated connections, labeled C, D, G and H in Figure 1), for simplicity these are not represented in Figure 3. Bias units are only used in one experiment, as indicated.

**Figure 4:**
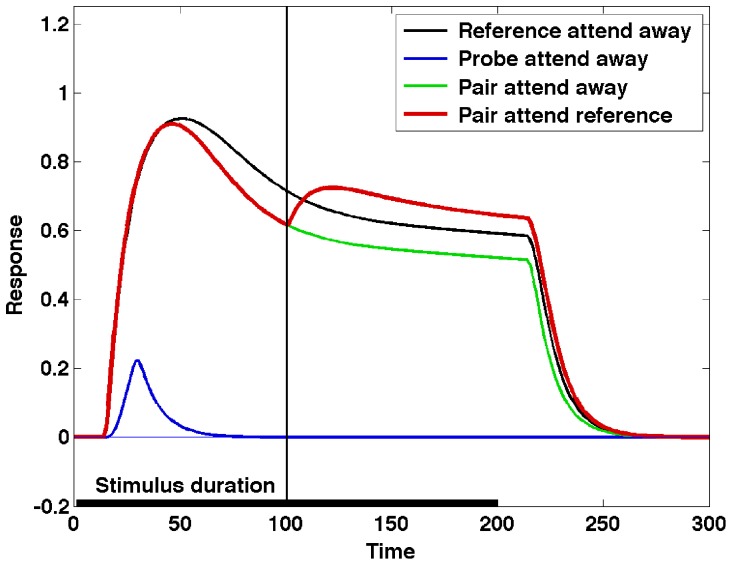
The output of neuron E1,2 in the four experimental conditions. The “Pair attend reference” (red) line represents the condition when the reference stimulus is attended (i.e. neuron E1,2 wins the top-level θ-WTA).

The model network is tested in the same four conditions as the Reynolds et al. experiment, and the results are presented using the same color-coding as that used in Figure 2. Figure 4 represents the output of neuron E_1,2_ in these four experimental conditions. The “Pair attend reference” (red) line represents the condition when the reference stimulus is attended (i.e. neuron E_1,2_ wins the top-level θ-WTA). The attentional selection process is triggered 100 ms after the presentation of the stimulus, indicated by a vertical line. It can be observed that the response for the unattended pair of stimuli is lower than the response for the reference stimulus alone, and that attending to the reference stimulus in the pair enhances the neuron's response. The relative responses in the different conditions can be changed by manipulating the weights of the different connections, as illustrated in Appendix S2.

Certain characteristics of the response, such as the amount of attentional modulation and the timing of the effect, can be manipulated to provide further insight into the various modes of operation possible in ST.

**Figure 5:**
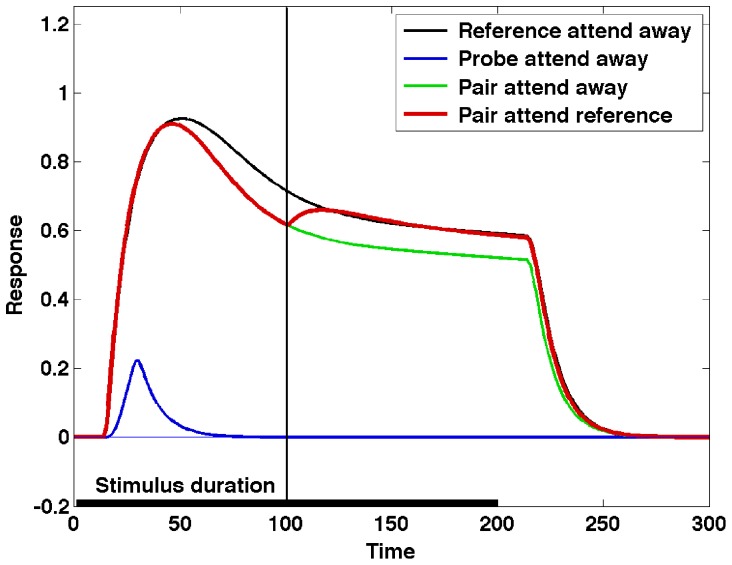
Gating is reduced to 50%. Experiments show that the amount of attentional modulation depends on a number of factors. Here we illustrate the effect of reducing the range of the gating signal, resulting in an attended response that more closely matches the reference alone condition.

Experiments show that the amount of attentional modulation depends on a number of factors, including area studied and target-distractor similarity (see [27] for a summary). Figure 4 has been obtained by restricting the gating signal *γ_k_* to the values of 0 (unattended) and 1 (attended), resulting in maximum attentional modulation, but by reducing the range of the gating signal, the ST equations can provide control over the modulation. For example, in Figure 5, the amount of inhibition for the unattended stimulus is only 0.5, resulting in an attended response that more closely matches the reference alone condition.

The control of this variable gating effect is not included in the equations, but since gating is controlled by the θ-WTA, and the θ-WTA depends of target-distractor similarity, it is not implausible to hypothesize that the θ-WTA process also controls the magnitude of the gating, but the mechanism is unknown.

**Figure 6:**
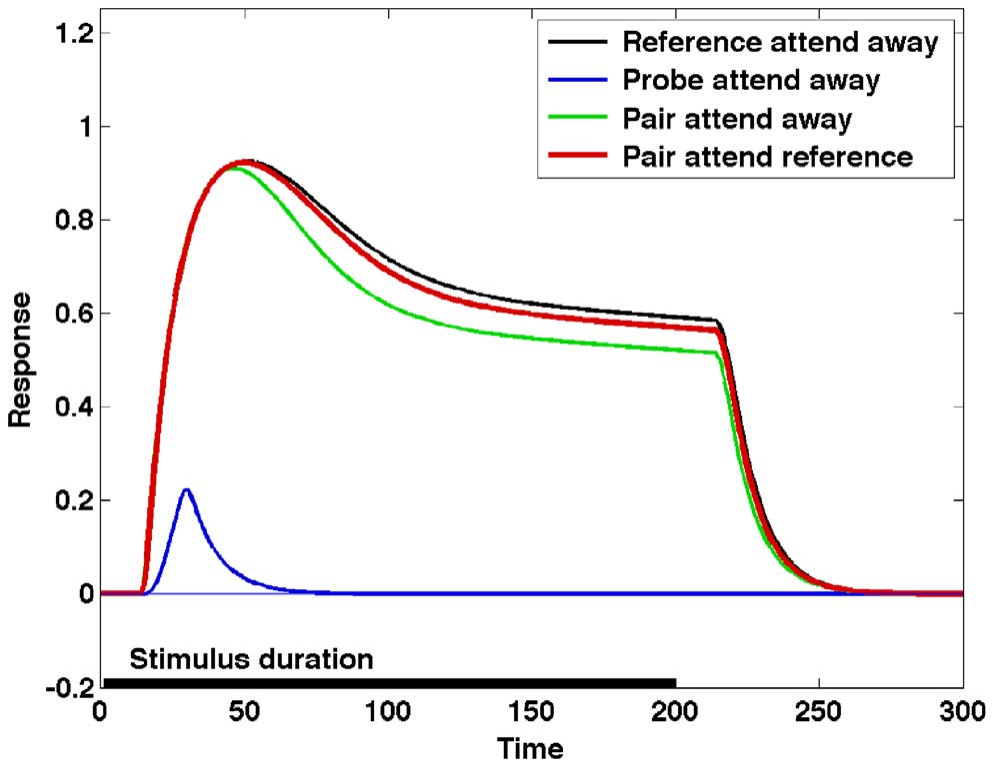
The effect of spatial cueing. Including a spatial bias towards the reference stimulus in the ST model (the equivalent of spatial cueing prior to stimulus presentation), shows another mode of operation made possible by the ST equations.

One significant difference between the simulation results presented above and the Reynolds et al. experiment is that the location of the target has been cued in the experiments, but not in the model. Including a spatial bias towards the reference stimulus in the ST model, which is the equivalent of spatial cueing prior to stimulus presentation, shows another mode of operation made possible by the ST equations. The response of the model in the four conditions with pre-cueing of the reference stimulus is shown in Figure 6. The effect of cueing in ST has been described in detail and experimentally investigated [55].

An interesting characteristic of the ST response in Figures 4 and 5 is the strong rebound of the neural response to the pair of stimuli when the selection process is triggered. The rebound is (at least in part) due to the fact that in the model gating is applied instantaneously, resulting in a transient in the input signal to the neuron. This transient produces a strong change in the response, similar to the one corresponding to the stimulus onset. It is possible to control the transient, and thus the rebound, by applying gating gradually, which is realistic, as it is the result of the activation of the gating neurons.

Assuming that the rebound corresponds to a real phenomenon, can we predict the kinds of experiments that will show a similar effect? The Reynolds et al. experiment does not show any obvious equivalent, possibly due to the spatial cueing, as discussed above. The network discussed so far contains a single layer of neurons. In more realistic multi-layer networks, the results of top-level competitions take time to propagate back through the network, resulting in the attentional modulation being applied at different times in different layers. This means that under the right conditions, not only will attentional modulation show the rebound, but since at each different layer the corresponding rebound will be produced at a different time, the modulation at higher levels of the network will show a pattern of attentional modulation consistent with the accumulation of lower level rebounds.

### Latency of Attentional Modulation in a Processing Hierarchy

One of the early predictions of ST is that of a temporal ordering and thus a time course of hierarchical modulation effects. Specifically, modulation will be seen at the highest levels first and at the lowest levels last, the opposite of what all other models would suggest. This is a strong differentiator between ST and the other models described above. Selection of the strongest response at the top of a hierarchical network triggers a recurrent localization process. At each successive layer of recurrence, part of the input to the selected neurons is suppressed, leading to a change in response for all neurons upstream. The changes in response thus occur in time steps defined by the number of layers in the pathway downward, plus the time it would take for the effect of suppression at one layer to ripple up to the neuron being examined. Significant experimental evidence for this prediction has been presented - e.g. [12], [56]–[60]. This model allows us to strengthen STs suppressive surround prediction – the imposition of the suppressive surround also has the same latency pattern. That is, a spatial suppressive surround due to the recurrent localization component of ST, will be observed layer by layer in a top-down order. To date, the surround has only been observed in area V1 [59] at the expected time (250 ms after stimulus onset) and experiments that test the existence of the surround in higher order areas have not been yet performed.

One of the earliest experiments to study the timing of selective attention modulation across areas of the macaque visual system [12], was performed by simultaneous recordings from different areas, thus allowing direct comparison of the magnitude and timing of the responses and modulation. Recordings of laminar event-related potential and current source density response profiles were sampled with linear array multicontact electrodes. The subjects were required to perform alternative discrimination tasks on auditory and visual stimuli, while ignoring the stimuli in the other modality. The visual stimuli were diffuse light flashes differing in intensity or color presented at the fixation point, and the effect of attention was evaluated by comparing responses to the visual stimuli when attended vs. when ignored.

Responses were summed over all contacts at each time point to obtain a sum average rectified current flow (sAVREC), while the difference between the ignored and attended conditions was summed to obtain the difference average rectified current flow (dAVREC). The temporal evolution of the responses and of the attentional modulation was determined by comparing sAVREC and dAVREC in each of the investigated areas. Of interest in this context is the finding that the earliest attentional modulation (i.e. the earliest significant dAVREC) was observed in the highest areas, and progressively later towards the lower areas.

### Latency of Attentional Modulation in ST

**Figure 7:**
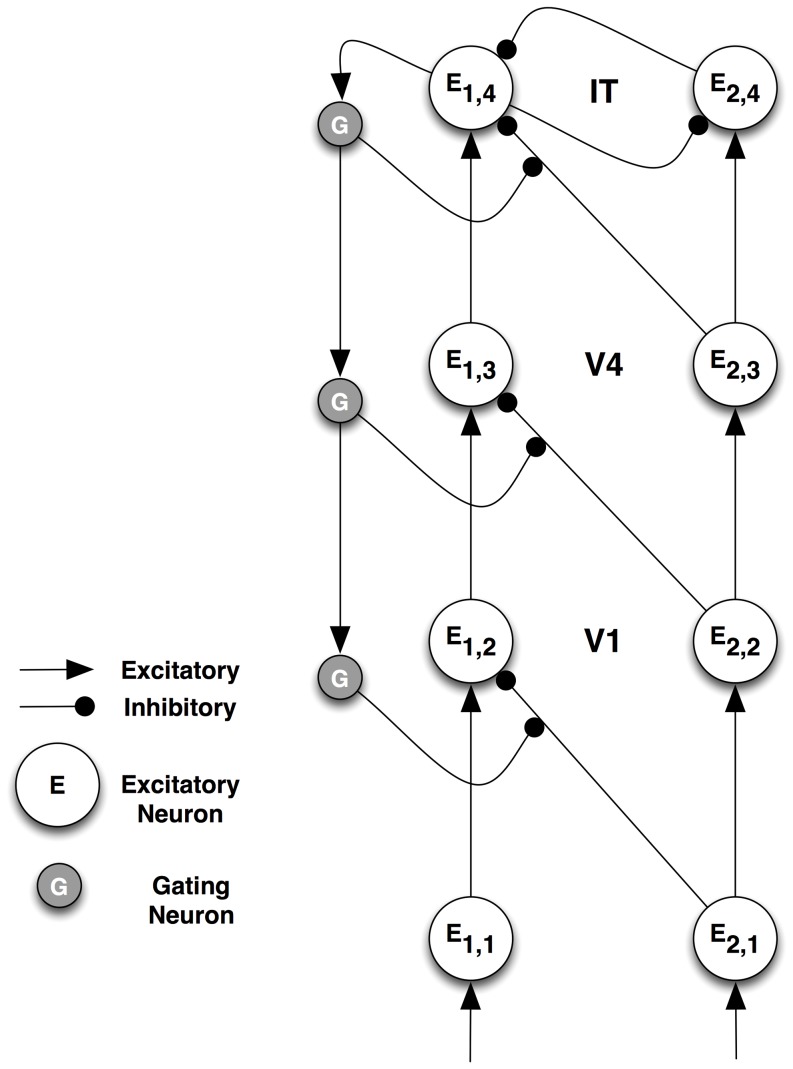
Full network for detailed timing analysis. To investigate the timing of the effects in a ST hierarchy, the circuit described in Figure 3 was replicated to form four layers, with the output of one processing layer driving the input of the next. To illustrate the top-down nature of the ST process, gating control units are shown on the left side of the hierarchy. The circuit is symmetrical, and gating control units exist for each connection, but are omitted for clarity (same for the inhibitory interneurons). The top-level θ-WTA process (indicated by the mutually inhibitory connections at the top level of the network) determines a winner, and the corresponding gating signals are propagated down the network, triggering local θ-WTA processes within each winning neuron's afferents.

**Figure 8:**
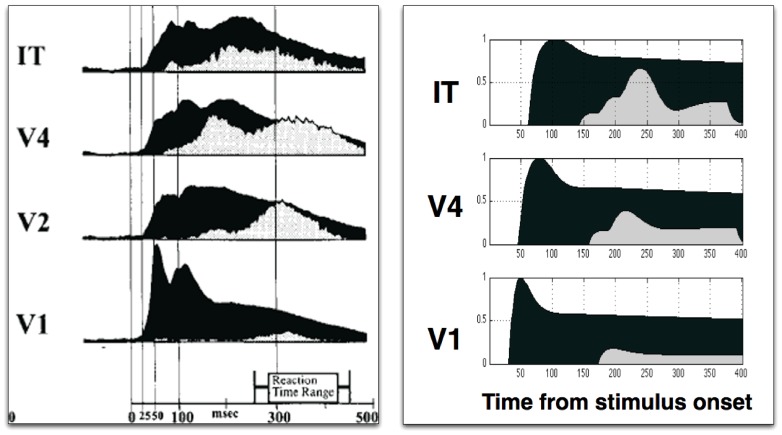
Attentional modulation of responses. Black indicates the neural response to the stimulus, while the attentional modulation is represented in grey. (a) Temporal pattern of activations and attentional modulation in single-unit recordings in primates performing attentional tasks. Adapted from Fig. 9b in [12]. The neural activation (in black) shows the responses being generated progressively later in more superior areas, while the attentional modulation (grey) appears earlier in superior areas and later in early areas. (b) Model results showing a similar activation and modulation temporal pattern.

To investigate the timing of the effects in a ST hierarchy, the circuit described in Figure 3 was replicated to form four layers, as shown in Figure 7, with the output of one processing layer driving the input of the next. To illustrate the top-down nature of the ST process, gating control units are shown on the left side of the hierarchy. The circuit is symmetrical, and gating control units exist for each connection, but are omitted for clarity (same for the inhibitory interneurons). The neurons are characterized by the same differential equations introduced above. Stimuli are presented at the bottom and the activations propagate through the network – the black curves in Figure 8. The top-level θ-WTA process determines a winner, and the corresponding gating signals are propagated down the network, triggering local θ-WTA processes within each winning neuron's afferents. This results in the modulation of the neural responses, as described above.

Figure 8 compares the model spiking rates on the right with average transmembrane currents recorded from neurons in different visual areas in the attention experiment described in the previous section on the left, for the neurons corresponding to the attended stimulus. The figures compare the relative timing of the initial response (in black) and the attentional modulation (in gray) across visual areas. The attentional modulation for the experiment is dAVREC, and similarly, for the model it is difference between the responses of the interpretive units in the ignored and attended conditions.

**Figure 9:**
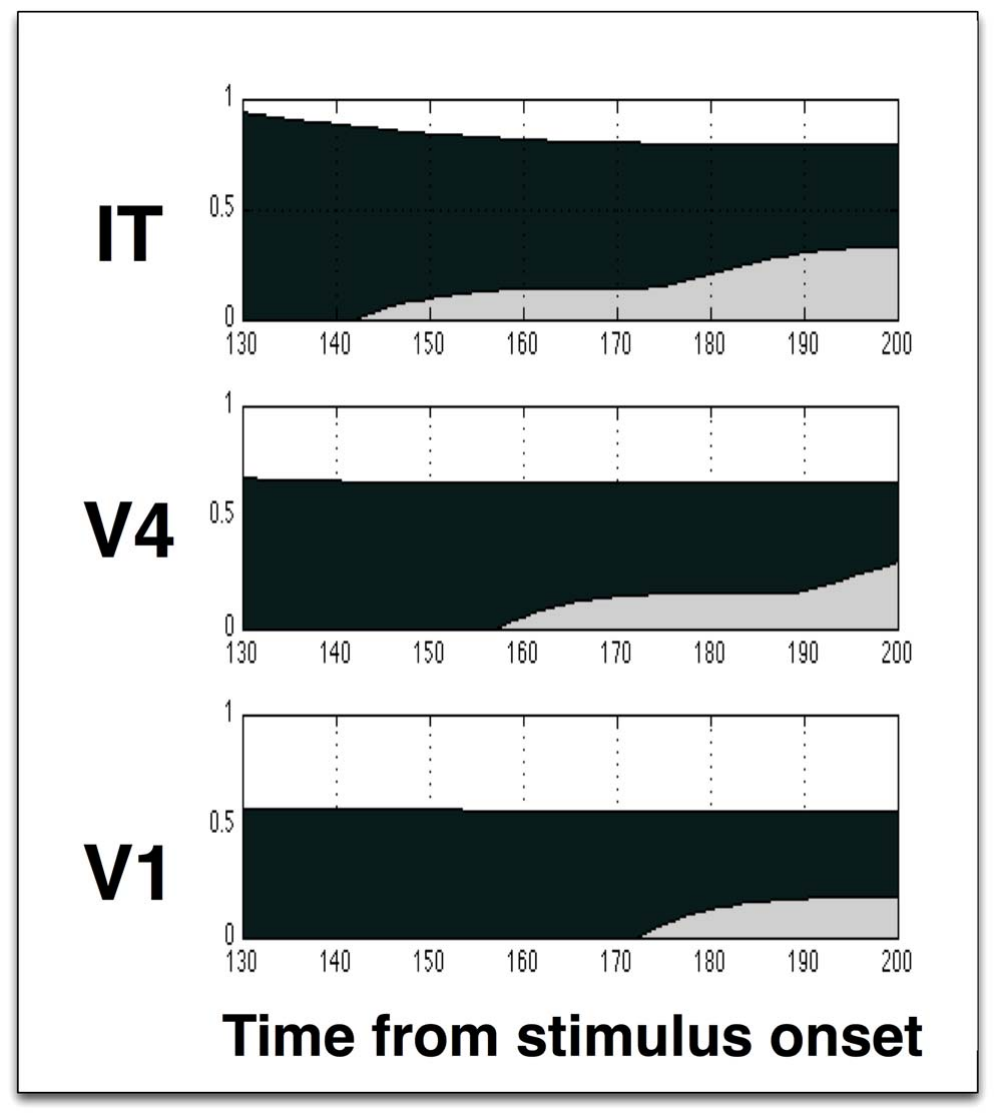
Model results - detail on the 130–200 ms interval. The temporal pattern of attentional modulation, with earlier modulation of superior visual areas, is visible.

A detailed representation of the relevant 130–200 ms time interval is presented in Figure 9. The neural activation (in black) shows the responses being generated progressively later in more superior areas, while the attentional modulation (grey) appears earlier in superior areas and later in early areas. In the model, the propagation time between visual areas has been set to 15 ms, for both the feedforward and the feedback stage. Mehta et al. do not provide a quantitative evaluation of the delays, but this could easily be integrated into the model. Note that the modeling is qualitative, meant to show only the general timings and shape of the response and modulation, as the details of the real network are unknown, however, the similarity to the macaque data [12] is striking, and a key characteristic of attention not found in other models.

## Discussion

We have shown through computational modeling that ST produces qualitatively equivalent modulatory effects for single neurons, similar to other models, but in addition qualitatively correct results for a hierarchy of neurons in contrast to other models, without the need for external attentional or bias inputs. Other models encode attention by modifying contrast, bias or gain parameters whose value changes from attended to unattended values. These models are all silent on how this value is set or how selections may occur whereas ST has an integrated selection mechanism. All except for ST are data-fitting models and unable to accept image input and produce the required behavior as ST can. The most immediate first impression of comparison is how different all the formulations appear. ST, the biased competition model [19], reentry hypothesis [22] and predictive coding/biased competition [35], [36] are based on the firing rate neuron formulation; feature similarity gain [23]–[25], normalization model of attention [30], normalization model of attentional modulation [31] are divisive contrast normalization models; the cortical microcircuit for attention model [32] and the integrated microcircuit model of attentional processing [33] are spiking neuron models; the neurodynamical model [21] employs mean-field approximation. The biased competition model [19], feature similarity gain [23]–[25], normalization model of attention [30], and normalization model of attentional modulation [31] are single-neuron models. The normalization model of attention [30] goes beyond a single neuron in that it takes larger visual fields into account. The Cortical Microcircuit for Attention model [32], the neurodynamical model [21], the reentry hypothesis [22], predictive coding/biased competition [35], [36] and ST employ networks of several types neurons. The neurodynamical model [21], the reentry hypothesis [22], predictive coding/biased competition [35], [36] and ST operate over complex network architectures. But this seems to be more of a feature of the model scope and starting assumptions than of substance.

As a first point of comparison, all of the models except for ST are data-fitting models. Each would take existing data and determine parameter values of a set of equations that provide the closest fit to the data. As such, equations with high degrees of freedom (most variables) and nonlinearities have the greatest potential to capture the data presented. They also are the least specific or have the least scientific value because a high-enough number of variables and nonlinearities may capture just about any data set. ST takes input images and determines responses to that input, a completely different approach because the data and/or behavior must be produced for specific input. Again, a computer program may behave in any manner its programmer sees fit; it too may have suspect scientific value unless it has been developed on a sound and principled theoretical foundation. The development of ST has been conducted on such a sound theoretical foundation, and all aspects of its realization have been guided by it [9].

The next dimension along which these models may be compared is the manner in which attention is incorporated. The cortical microcircuit for attention model [32] encodes attention by modifying a linear contrast parameter. Similarly, the biased competition model [19], feature similarity gain [23]–[25], the normalization model of attention [30], and the normalization model of attentional modulation [31] all provide a single parameter that controls attention; this is a bias or gain whose value changes from ‘attended’ to ‘unattended’ values. For example, in the biased competition model [19], attention is implemented by increasing by a factor of 5 both excitatory and inhibitory synaptic weights projecting from the input neuron population responding to the attended stimulus. In the normalization model of attentional modulation [30] there is a parameter that takes values equal to 1 for unattended stimuli and larger for attended ones. Its effect is multiplicative; it multiplies the product of slope of normalization and contrast in the exponent of the response function. These models are all silent on how this value is set or how selections may occur. The cortical microcircuit for attention model [32] is also silent in this regard. In the integrated microcircuit model of attentional processing [33] the self-sustained activity of an additive gating signal is triggered by the presentation of the stimulus to be attended during a cueing interval. This allowed the investigation of the effect of attention on the baseline activity of neurons. In the neurodynamical model [21], processing can be controlled by external task signals that select either neuron pools associated with an object to be searched, or a location, in order to identify the object at that location. In the reentry hypothesis [22], object recognition neurons with RFs covering the visual area of interest have their sensitivity and gain increased by reentrant signals from movement areas, thus having an advantage in the competition process. Predictive coding/biased competition [35], [36] relies on feedback signals that can originate from node activations calculated at higher-levels in the hierarchy and/or external inputs, and attentional feedback is treated exactly the same as feedback from higher stages in the hierarchy. Although these models all give the expected, non-attentive, results without this attention parameter, they cannot demonstrate attentive effects without some external setting of this parameter. However, in the natural evolution of attentional modeling, it is clearly of interest to provide a solution to this setting as well, thus removing what is otherwise a serious model limitation. ST does exactly that, providing an explicit algorithm for how this parameter is derived, a method that is applicable to any model without such an explicit computation.

Different forms of competition have been employed by different authors. Most models implement competition through the mutual inhibition between neural outputs, e.g. the biased competition model [19], the neurodynamic model [21], and the reentry hypothesis [22]. In a few cases the competition involves the inputs, in the form of output neurons suppressing the input of other neurons, e.g. predictive coding/biased competition [35], [36], or a direct competition between the inputs in ST.

## Conclusions

In this paper we have shown that ST generates patterns of attentional modulation and its temporal progression. The selection mechanism employed is completely integrated within the basic equations, without the need for external attentional signals. The selection can be aided by feature and location task biases, but these are not necessary. Further, it is consistent with the conclusions that attention can be best considered as modulating inputs to neurons, both spatial and feature [61]: ST's equations do exactly this, manipulating neural inputs to achieve the required attentive effectat a qualitative level of description. Most of the other models described are quantitative, that is, they can be quantitatively compared to actual neural recordings in terms of time and firing rates, whereas ST cannot. On the other hand, they cannot explain the top down latency of attentional modulation just like they cannot explain how attentional focus is determined. The transition of ST into a quantitative model is not an intellectual challenge; parameters derived from real data can be easily obtained and ST's basic equations modified appropriately. For the other models, easy transitions to deal with determination of focus and timing are not possible without whole-scale changes to the model. The reason for these differences is that ST was designed as first-principles model not relying on data as a starting point whereas the other models all are data-fitting models.

The Selective Tuning theory requires that neural connections from higher to lower order visual areas, usually not differentiated in other models, comprise several different functionalities. Within the model, connections that convey bias signals, gating control signals and suppressive feedback signals are explicit. Bias is what is seen in most other models as the attention parameter but in ST these provide suppressive signals to task-irrelevant neurons, the benefit being that those neurons would respond less to any input signals thus not creating strong interfering signals for the stimuli of interest (i.e., assist in improving signal to noise between stimuli of interest and background). The gating control signals provide a timing signal that initiates the overall recurrent process in the correct order. In an important sense, these may be considered as a possible source of low frequency oscillation because attention has a cyclic behaviour in the ST context (as reported by [62] and predicted by [39]). The suppressive feedback signals, which we also can term attentive recurrence, implement the bound component of the Branch-and-Bound algorithm responsible for the top-down tracing of neural connections from high level attended neurons to stimulus source. In the broader visual processing context within which ST operates, other forms of non-feedforward connectivity are also included (see [9]), such as lateral inhibition within a visual area as well as between visual areas, local feedback between hierarchically adjacent areas, and more. Although the existence of winner-take-all circuits has been previously shown as mentioned earlier, our particular variant, with the inclusion of a threshold on competition, seems a natural extension but still requires verification. In order to further refine these predictions both theory and experiment are critical as only close collaborations between theory and experiment will reveal the ways in which to further develop our models.

## Supporting Information

Figure S1 The effect of changing the weight of the excitatory input for the preferred stimulus. Excitatory input for preferred stimulus changed between 0.8 and 0.5, while the others are fixed at the default value.
(TIF)

Figure S2 The effect of changing the weight of the inhibitory input for the preferred stimulus values. Inhibitory input for preferred stimulus changed between −0.3 and −0.6, while the others are fixed at the default value.
(TIF)

Figure S3 The effect of changing the weight of the excitatory input for the preferred stimulus. Excitatory input for non-preferred stimulus changed between 0.1 and 0.4, while the others are fixed at the default value.
(TIF)

Figure S4 The effect of changing the weight of the excitatory input for the preferred stimulus. Inhibitory input for non-preferred stimulus changed between −0.2 and −0.5, while the others are fixed at the default value.
(TIF)

Appendix S1 Computational details of the models of attentive neural modulation presented in the paper.
(DOCX)

Appendix S2 Parameters used in the simulations presented in the paper. A discussion of the effect of changing these parameters on the behavior of the model.
(DOCX)
