# Supplementary figures and images for: Attentional Modulation and Selection – An Integrated Approach
Source: PLoS One. 2014 Jun 25;9(6):e99681. doi: 10.1371/journal.pone.0099681 (PMC4070899; doi:10.1371/journal.pone.0099681)

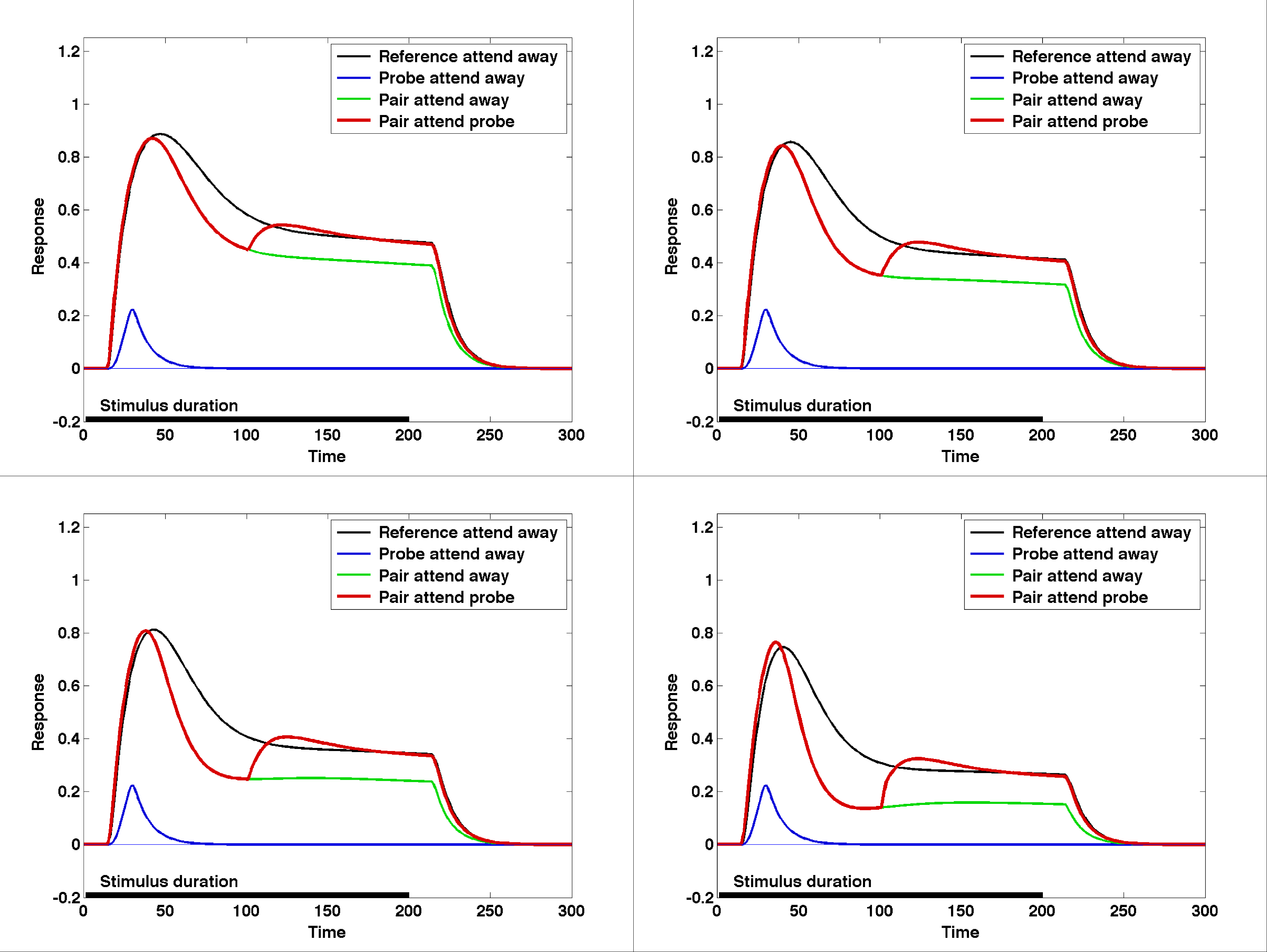

Supplement: Figure S1 — The effect of changing the weight of the excitatory input for the preferred stimulus. Excitatory input for preferred stimulus changed between 0.8 and 0.5, while the others are fixed at the default value. (TIF) [file pone.0099681.s001.tif]

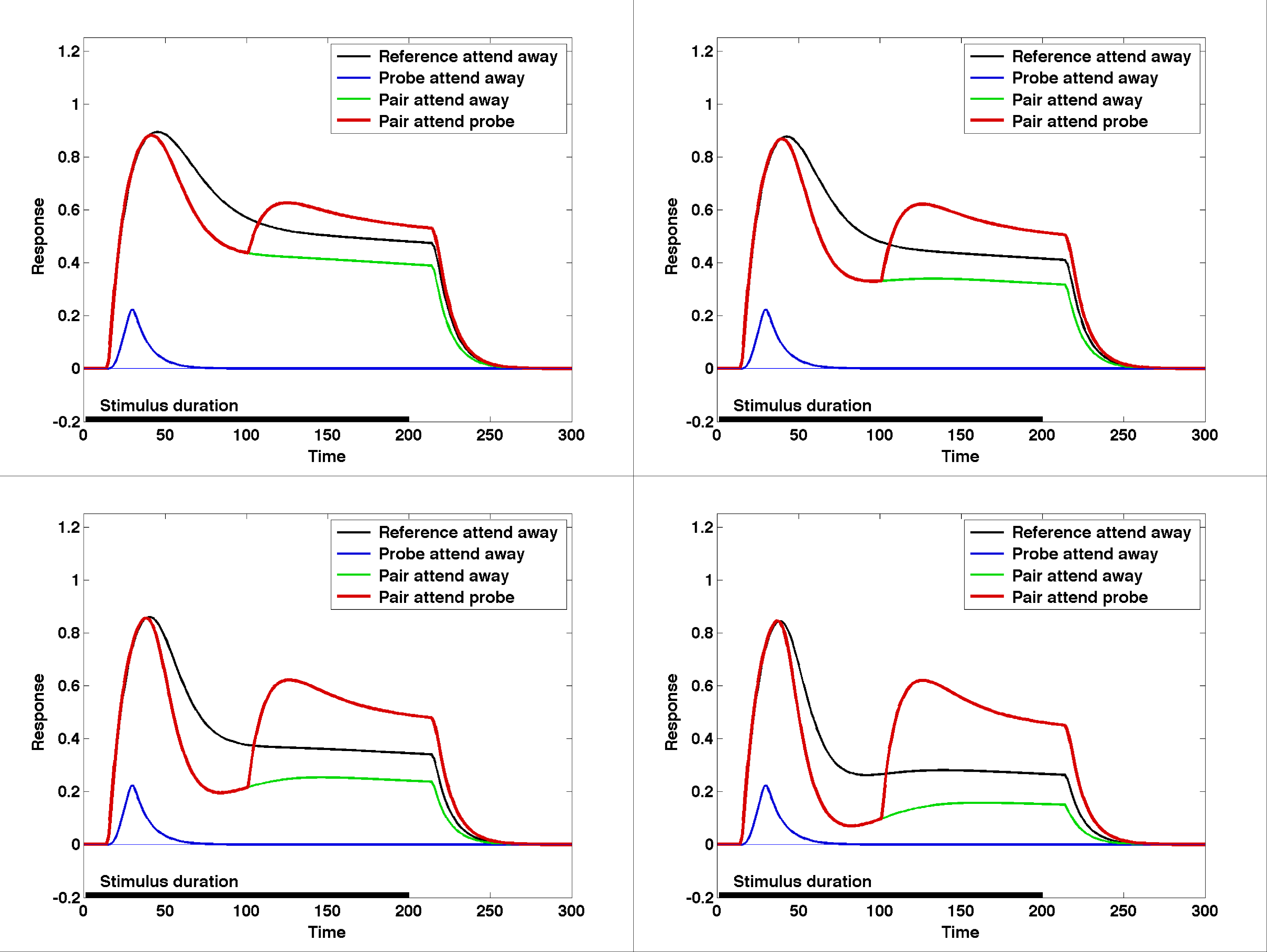

Supplement: Figure S2 — The effect of changing the weight of the inhibitory input for the preferred stimulus values. Inhibitory input for preferred stimulus changed between −0.3 and −0.6, while the others are fixed at the default value. (TIF) [file pone.0099681.s002.tif]

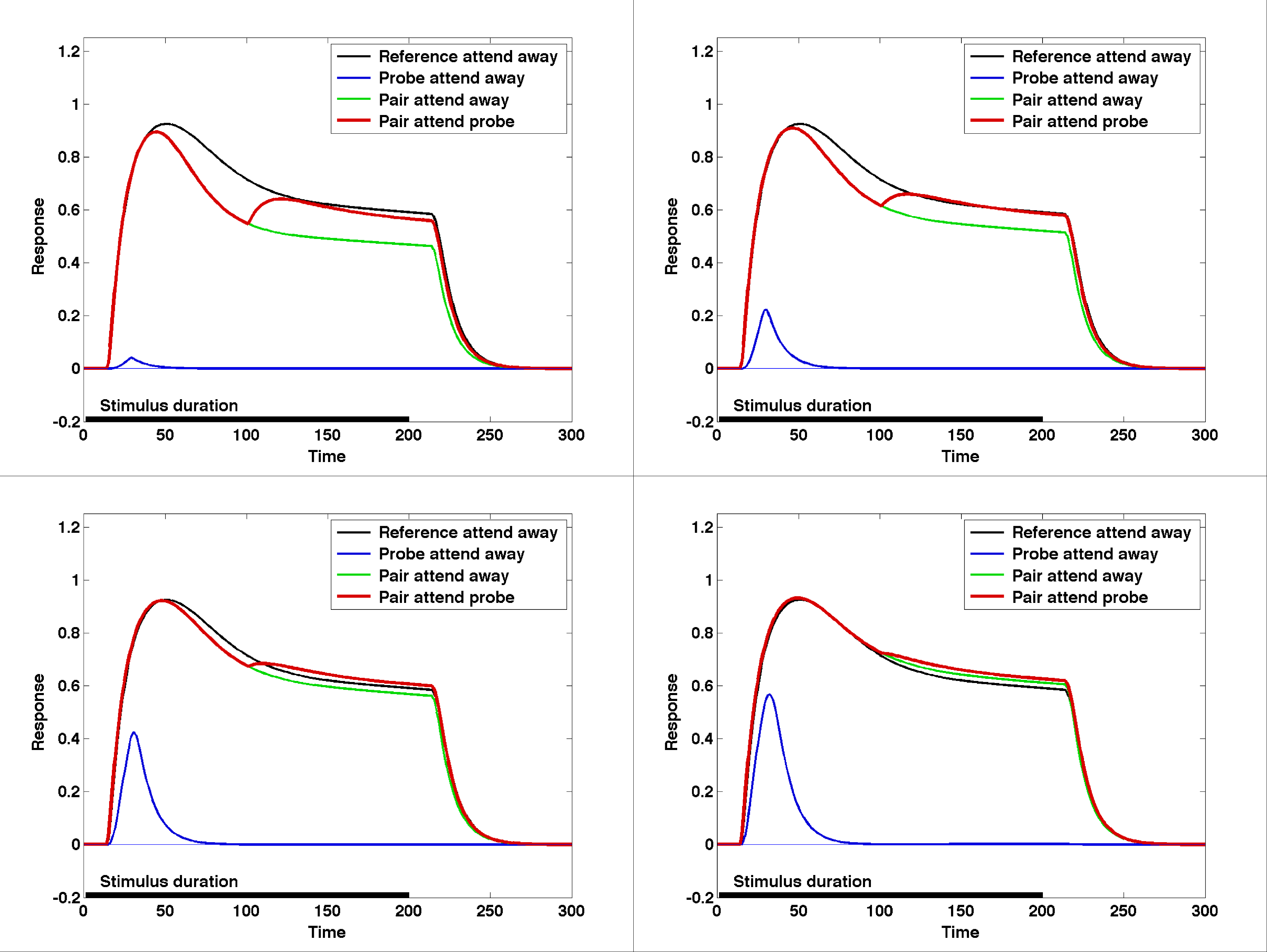

Supplement: Figure S3 — The effect of changing the weight of the excitatory input for the preferred stimulus. Excitatory input for non-preferred stimulus changed between 0.1 and 0.4, while the others are fixed at the default value. (TIF) [file pone.0099681.s003.tif]

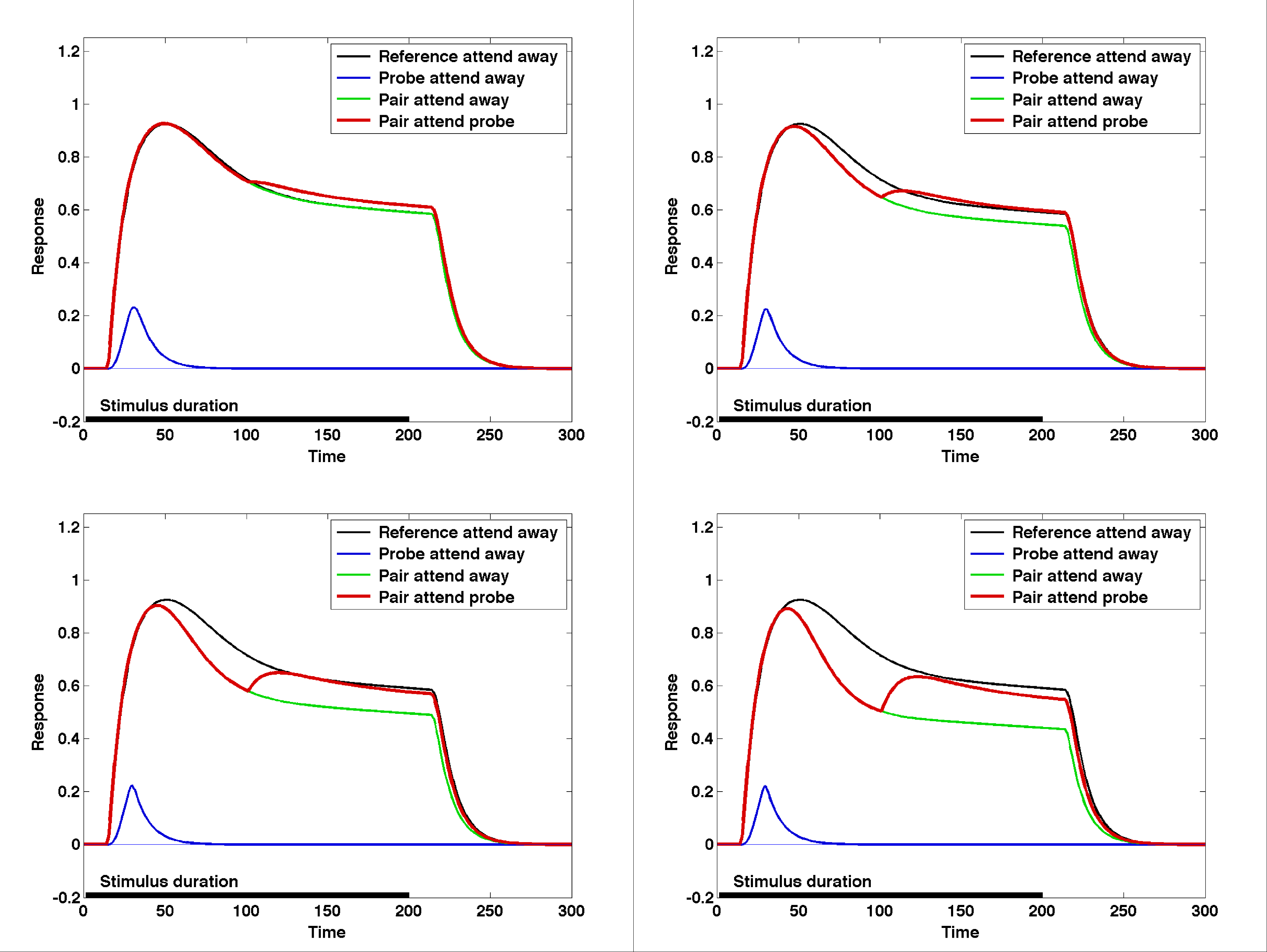

Supplement: Figure S4 — The effect of changing the weight of the excitatory input for the preferred stimulus. Inhibitory input for non-preferred stimulus changed between −0.2 and −0.5, while the others are fixed at the default value. (TIF) [file pone.0099681.s004.tif]
