# Supplementary material for: Attentional Modulation and Selection – An Integrated Approach
Source: PLoS One. 2014 Jun 25;9(6):e99681. doi: 10.1371/journal.pone.0099681 (PMC4070899; doi:10.1371/journal.pone.0099681)
Supplement: Appendix S1 — Computational details of the models of attentive neural modulation presented in the paper. (DOCX) [file pone.0099681.s005.docx]

Appendix S1

Models of Attentive Neural Modulation – Computational Details

The descriptions and equations included below are meant mainly to illustrate the wide variety of solutions proposed, and to highlight the way attentional modulation is implemented, rather than being exhaustive descriptions of the models. For full details, complete sets of equations and biological justification, the reader is referred to the original sources.

The Biased Competition model [19] has been proposed as a demonstration of the biased competition theory. The temporal evolution of a neuron’s firing rate is given by:

$$\frac{dy}{dt}=(B-y)E-yI-Ay$$

where $E=x_{1}w_{1}^{+}+x_{2}w_{2}^{+}$ is the excitatory input for two stimuli within the same receptive field, and$I=x_{1}w_{1}^{-}+x_{2}w_{2}^{-}$ the inhibitory input for two stimuli within the same receptive field. The *w*'s are positive and negative synaptic weights. The equilibrium response is described by , where *B* is the maximum response and *A* is a decay constant. Attention is assumed to increase the strength of the signal coming from the inputs activated by the attended stimulus, implemented by increasing the associated synaptic weights.

The Neurodynamical model [21] presents a large-scale implementation of biased competition that consists of several interconnected network modules simulating different areas of the dorsal and ventral path of the visual cortex. Each module consists of a population of cortical neurons arranged in excitatory and inhibitory pools. The temporal evolution of the system is described within the framework of a mean-field approximation, i.e. an ensemble average of the neural population is calculated in order to obtain the corresponding activity. For example, the current activity of the excitatory pools in the posterior parietal (PP) module is given by:

$$\tau\frac{\partial}{\partial t}I_{ij}^{PP}\left(t\right)=-I_{ij}^{PP}\left(t\right)+aF\left(I_{ij}^{PP}\left(t\right)\right)-bF\left(I^{PP,I}\left(t\right)\right)+I_{ij}^{PP-V1}\left(t\right)+I_{ij}^{PP,A}+I_{0}+v$$

where $I_{ij}^{PP,A}$ is an external attentional spatial-specific top-down bias, $I_{0}$ is a diffuse spontaneous background input, $\upsilon$ is Gaussian noise. The intermodular attentional biasing $I_{ij}^{PP-V1}$ through the connections with the pools in the module V1 is:

$$I_{ij}^{PP-V1}\left(t\right)=\sum_{k,p,q,l} W_{pqij}F\left(I_{kpql}^{V1}\left(t\right)\right)$$

and the activity current of the common PP inhibitory pool evolves according to:

$$\tau\frac{\partial}{\partial t}I^{PP,I}\left(t\right)=-I^{PP,I}\left(t\right)+c'\sum_{i,j} F\left(I_{ij}^{PP}\left(t\right)\right)-dF\left(I^{PP,I}\left(t\right)\right)$$

Similar equations govern the dynamics of the other models.

The Feedback Model of Visual Attention [20] improves on the Biased Competition Model by providing a biologically-justified mechanism and microcircuitry for input modulation. The key observation that drives the model is that feedforward connections seem to be primarily made in basal dendrites, while feedback connections preferentially target apical dendrites, thus appearing to have functionally different roles. The activations of the apical and basal dendrites are given by:

$y_{jk,apical}^{t}=\sum_{i=1}^{m_{a}} v_{ijk}x_{ijk}^{t-1}$ and $y_{jk,basal}^{t}=\sum_{i=1}^{m_{b}} w_{ijk}X_{ijk}^{t-1}$

where *j* and *k* are indices used to localize the neuron in the network, *m_a_* and *m_b_* are the total number of apical and basal synapses, respectively, *v_ijk_* and *w_ijk_* are synaptic weights associated with the input from neuron *i* (apical and basal, respectively), *x* is the output of neuron i. The apical inputs originate from higher cortical regions or are top-down signals from outside the model. The basal inputs are the outputs of neurons in lower cortical regions, after pre-integration lateral inhibition:

$$X_{ijk}^{t}=x_{ijk}^{t}\left\{1-\alpha^{t}\max_{\begin{matrix} p=1 \\ \left(p\neq j\right) \end{matrix}}^{n} \left[\frac{w_{ipk}}{\max_{q=1}^{m}\left(w_{qpk}\right)}\frac{y_{pk}^{t-1}}{\max_{q=1}^{n}\left(y_{qk}^{t-1}\right)}\right]\right\}^{+}$$

where α^t^ scales lateral inhibition, $y_{pk}^{t-1}$ is the output of neuron p in region k at the previous time step, and $\left\{z\right\}^{+}$ the positive half-rectified value of $\left\{z\right\}$. The Feedback Model of Visual Attention proposes that feedback activations multiplicatively modulate the total feedforward activation:

$y_{jk}^{t}=y_{jk,basal}^{t}\left(1+y_{jk,apical}^{t}\right)$ .

The reentry hypothesis [22] models top-down modulation as a gain control mechanism on the input feedforward signal, increasing activations that match top-down predictions. Considering two interconnected areas I and II, the generic formulation of the modulation of the input signal $I_{d,k,x}^{II}$ to area II combines the filtered feedforward input $F\left(r_{d,i,x'}^{I}\right)$ from area I with the summed top-down signal $\hat{r}_{d,i,x'}^{\gamma}$ where *γ∈{L,F}* is the origin of the attentional signal (*L* for location, *F* for feature):

$$I_{d,k,x}^{II}=w^{\uparrow}\cdot f\left(F\left(r_{d,i,x'}^{I}\right)\right)+\sum_{\gamma\in\left\{L,F\right\}} \left(A-r_{d,k,x}^{II}\right)_{+}\cdot f\left(F\left(r_{d,i,x'}^{I}\right)\cdot\hat{r}_{d,i,x'}^{\gamma}\right)$$

The nonlinear pooling function *f* defines the influence of the filtered afferents *F* on cell *k*. This generic equation is adapted to the specific connectivity of each area.

In the computational model of feature similarity gain proposed by [25], the neural response is described as a divisive contrast normalization process between the sum of squared linear responses to each stimulus component and the sum of squared contrasts plus a semi-saturation constant *σ* by:

$$H(x_{i},c_{i})=\frac{\sum_{i} (c_{i}F(x_{i}))^{2}}{\sum_{i} c_{i}^{2}+\sigma^{2}}$$

where each component that contributes to the activation has its own feature *x_i_* and contrast *c_i_*. The normalized firing rate *H* is modulated by a gain factor *G(y)* that has a tuning function similar to the stimulus-driven tuning function of that neuron, where *y* is the attended feature, resulting in a modulated response given by :

$R(x_{i},c_{i},y)=G(y)\left[H(x_{i},c_{i})+\delta\right]$ .

The parameter *δ* is the inherent baseline-firing rate of the neuron. The gain factor *G(y)* is greater than 1.0 for a preferred feature, and lower than 1.0 otherwise. The gain factor is a purely feature-based effect, and is independent of the spatial focus of attention and the properties of the visual stimulus.

Attentional modulation in saliency models is investigated in the context of object recognition [27]. A focus of attention spatial region is determined by means of center-surround competition along different feature dimensions and scales. A modulation mask is obtained by rescaling, smoothing and normalizing the focus of attention to the resolution of the layer where attention is to be applied (corresponding to visual areas V1 or V4)

If *S(x,y)* is the neural activity at position *(x,y)*, the modulated activity is computed according to

where 0≤μ≤1 is the strength of the attentional modulation. Modulation factors of 0.2-0.4 are shown to be sufficient for maximizing recognition performance in a multi-stimulus experimental setup.

The Normalization Model of Attention [30] combines neural selectivity (termed “stimulus drive”) with an external “attention field” and a “suppressive field”, that pools activations corresponding to non-preferred stimulus and unattended locations, which is used as in normalization. The resulting firing rates are defined as:

$$R(x,\theta)=\left|\frac{A(x,\theta)E(x,\theta)}{S(x,\theta)+\sigma}\right|_{T}$$

where

$S(x,\theta)=s(x,\theta)*A(x,\theta)E(x,\theta)$.

*E(x,θ)* is the stimulus drive at location *x* for orientation *θ. A(x,θ*) is the attentional field, *S(x,θ)* is the suppressive drive. *σ* is a constant that determines the neuron's contrast gain. |...|*_T_* specifies rectification with respect to threshold *T.* ✴ denotes convolution, *s(x,θ)* gives the extent of pooling. Stimulus contrast is also included in the equations and is not shown here; see [30] for further details. *A(x,q*) *=1* everywhere except for the positions and features that are to be attended, where *A(x,q) >1.*

The Normalization Model of Attentional Modulation [31] proposes that the primary effect of attention is to modulate the strength of normalization mechanisms. With two stimuli present in the RF, the neuron’s firing rate is given by:

$$R_{1,2}=\left[\frac{N_{1}\cdot(I_{1})^{u}+N_{2}\cdot(I_{2})^{u}}{N_{1}+N_{2}}\right]^{1/u}$$

where *N_i_* is the normalization term for each stimulus, *I_i_* is the direct input driven by each stimulus, and *u* is a power term that enables the modeling of different nonlinear summation regimens. Each normalization term depends on the contrast of the associated stimulus as:

$$N_{attended}=(1-s)(1-e^{-\beta\alpha c})+s$$

where *β*=1 for unattended item, *β* >1 for attended items, α is the slope of normalization, *c* is stimulus contrast; *s* is the baseline of normalization.

In the Cortical Microcircuit for Attention model [32], attention was modeled as a change in the driving current to the network neurons. Excitatory (E), feedforward interneurons (FFI) and top-down interneurons (TDI) are differentially modulated by attention: the firing rate of the FFI increases with spatial attention and decreases with feature-based attention, whereas the TDI increase their firing rate with feature-based attention and shift the network synchrony from the beta to the gamma frequency range. The neurons are arranged in columns, each containing neurons of the three types. The synaptic connectivity was designed to ensure inter-stimulus competition and the generation of cortical rhythms. The dynamics of each type of neuron are given by:

$$\begin{matrix} I_{E1}=I_{0,E1}+A_{E}(\beta_{E}c_{1}+(1-\beta_{E})c_{2}) \\ I_{FFI1}=I_{0,FFI1}+A_{FFI}(\beta_{FFI}c_{1}+(1-\beta_{FFI})c_{2}) \\ I_{E2}=I_{0,E2}+A_{E}(\beta_{E}c_{1}+(1-\beta_{E})c_{2}) \\ I_{FFI2}=I_{0,FFI2}+A_{FFI}(\beta_{FFI}c_{1}+(1-\beta_{FFI})c_{2}) \end{matrix}$$

where *A* is a scaling factor; *c* is the stimulus contrast; *β* is stimulus selectivity, *0.5 ≤ β < 1*; *I_0_* is a constant offset current.

The integrated microcircuit model of attentional processing [33] is composed of a reciprocally connected loop of two (sensory and working memory) networks. The channel kinetics are modeled by:

$$\frac{ds}{dt}=-\frac{1}{\tau_{s}}s+\alpha_{s}x\left(1-s\right)$$

$$\frac{dx}{dt}=-\frac{1}{\tau_{x}}x+\sum_{i} \delta\left(t-t_{i}\right)$$

where *s* is the gating variable, *x* is a synaptic variable proportional to the neurotransmitter concentration in the synapse, *t_i_* are the presynaptic spike times, τ*_s_* ms is the decay time of NMDA currents, τ*_x_* controls the rise time of NMDAR channels, and α_s_ controls the saturation properties of NMDAR channels at high presynaptic firing frequencies.

In the predictive coding/biased competition model [34], every processing stage consists of prediction and error detection nodes, with firing rate dynamics defined by:

$$\begin{matrix} e^{S_{i}}=y^{S_{i}-1}-\left(\mathbf{W}^{S_{i}}\right)^{T}y^{S_{i}} \\ y^{S_{i}}\leftarrow\left(1-\eta-\vartheta\right)y^{S_{i}}+\varsigma\mathbf{W}^{S_{i}}e^{S_{i}} \\ y^{S_{i}}\leftarrow y^{S_{i}}+\eta\left[\left(\mathbf{W}^{S_{i+1}}\right)^{T}y^{S_{i+1}}+\left(\mathbf{W}^{A_{i}}\right)^{T}y^{A_{i}}\right] \end{matrix}$$

where *S_i_* represents the current processing stage, *y^Si^* and *e^Si^* the vectors of predictive and error node activations, respectively, ***W*** are matrices of weight values, and *η*, *ς*, and *ϑ* are constant scale values. The external attentional modulation signal is *y^Ai^*, modulating activations through a set of weights ***W****^Ai^*.
