# Supplementary material for: Attentional Modulation and Selection – An Integrated Approach
Source: PLoS One. 2014 Jun 25;9(6):e99681. doi: 10.1371/journal.pone.0099681 (PMC4070899; doi:10.1371/journal.pone.0099681)
Supplement: Appendix S2 — Parameters used in the simulations presented in the paper. A discussion of the effect of changing these parameters on the behavior of the model. (DOCX) [file pone.0099681.s006.docx]

Appendix S2

**Methods**

The following values are used for the various parameters in the ST equations.

The exponent ξ= 3.0 represents a typical mean exponent for visual neurons [1].

The maximum firing rate is normalized to 1 (*Z*=1).

The base semi-saturation constant *σ_0_*= 0.8. The semi-saturation constant is modulated by fast and slow afterhyperpolarization currents, with *F_fast_* = 1.3, *F_slow_* = 2.

Time constant values (*τ* = 10ms, *τ_slow_* = 900ms , *τ _fast_* = 50ms) are consistent with physiological data [2,3].

As task bias is not used in these experiments, so *B* =1, except for the spatial cueing experiment presented in Figure 6, where a value of *B* =0.5 was used.

While all the constants used in this model are in the general range found in the visual cortex, they are not crucial to the results presented here. The same equations are used for all excitatory neurons.

The top level θ-WTA starts 100 ms after stimulus presentation, and θ is 20% of the maximum firing rate Z, i.e. 0.2.

The parameters that determine the attentional modulation in the first experiment are the four weights that feed into each top-level neuron, two excitatory and two inhibitory. The weights, given here with the default values used to produce Figures 4-6 are:

- pref_+_ = 1 - excitatory input for preferred stimulus. The lower the preferred excitation, the more effect the inhibitory inputs have, so the modulation is stronger.
- pref_−_ = −0.1 - inhibitory input for preferred stimulus. The stronger the preferred inhibition, the lower the initial response and the higher the rebound after ST.
- nonpref_+_ = 0.2 - excitatory input for non-preferred stimulus. The main effect is on the poor, attend away condition (green line). Higher excitation offsets distractor inhibition, so it reduces the effect of ST.
- nonpref_−_ = -0.35 - inhibitory input for non-preferred stimulus. The higher the inhibition, the stronger the modulatory effect of ST.

The circuit being symmetrical, the same four weight values are used for both top-level neurons. The effect of changing each weight independently is illustrated in Figure 10.

- Figure S1: pref_+_ - excitatory input for preferred stimulus, takes the values 0.8, 0.7, 0.6, and 0.5 (left to right, top to bottom). The lower the preferred excitation, the more effect the inhibitory inputs have, so the modulation is stronger.
- Figure S2: pref_−_ - inhibitory input for preferred stimulus, takes the values -0.3, -0.4, -0.5, and -0.6 (left to right, top to bottom). The stronger the preferred inhibition, the lower the initial response and the higher the rebound after ST.
- Figure S3: nonpref_+_ - excitatory input for non-preferred stimulus, takes the values 0.1, 0.2, 0.3, and 0.4 (left to right, top to bottom). The main effect is on the poor, attend away condition (green line). Higher excitation offsets distractor inhibition, so it reduces the effect of ST.
- Figure S4: nonpref_−_ - inhibitory input for non-preferred stimulus, takes the values -0.2, -0.3, -0.4, and -0.5 (left to right, top to bottom). The higher the inhibition, the stronger the modulatory effect of ST.

**References**

1. Sclar G, Maunsell JHR, Lennie P (1990) Coding of image contrast in central visual pathways of the macaque monkey. *Vision Research,* **30**(1), 1–10.

2. McCormick DA, Williamson A (1989) Convergence and divergence of neurotransmitter action in human cerebral cortex. *Proceedings of the National Academy of Sciences of the United States of America,* **86**(20), 8098–8102.

3. Sanchez-Vives MV, Nowak LG, McCormick DA (2000) Cellular mechanisms of long-lasting adaptation in visual cortical neurons in vitro. *Journal of Neuroscience,* **20**(11), 4286–4299.
